# Gum Kondagoagu/Reduced Graphene Oxide Framed Platinum Nanoparticles and Their Catalytic Role

**DOI:** 10.3390/molecules24203643

**Published:** 2019-10-09

**Authors:** Abhilash Venkateshaiah, Daniele Silvestri, Rohith K. Ramakrishnan, Stanislaw Wacławek, Vinod V. T. Padil, Miroslav Černík, Rajender S. Varma

**Affiliations:** 1 Department of Nanomaterials in Natural Sciences, Institute for Nanomaterials, Advanced Technologies and Innovation (CXI), Technical University of Liberec (TUL), Studentská 1402/2, 46117 Liberec 1, Czech Republic; abhilash.venkateshaiah@tul.cz (A.V.); daniele.silvestri@tul.cz (D.S.); rohith.kunjiparambil.ramakrishnan@tul.cz (R.K.R.); stanislaw.waclawek@tul.cz (S.W.); 2 Regional Centre of Advanced Technologies and Materials, Department of Physical Chemistry, Faculty of Science, Palacký University in Olomouc, Šlechtitelů 27, 78371 Olomouc, Czech Republic

**Keywords:** greener catalysts, kondagogu gum, RGO, Pt nanoparticle, 4-nitrophenol reduction

## Abstract

This study investigates an environmentally benign approach to generate platinum nanoparticles (Pt NP) supported on the reduced graphene oxide (RGO) by non-edible gum waste of gum kondagogu (GK). The reaction adheres to the green chemistry approach by using an aqueous medium and a nontoxic natural reductant—GK—whose abundant hydroxyl groups facilitate in the reduction process of platinum salt and helps as well in the homogenous distribution of ensued Pt NP on RGO sheets. Scanning Electron Microscopy (SEM) confirmed the formation of kondagogu gum/reduced graphene oxide framed spherical platinum nanoparticles (RGO-Pt) with an average particle size of 3.3 ± 0.6 nm, as affirmed by Transmission Electron Microscopy (TEM). X-ray Diffraction (XRD) results indicated that the Pt NPs formed are crystalline with a face-centered cubic structure, while morphological analysis by XRD and Raman spectroscopy revealed a simultaneous reduction of GO and Pt. The hydrogenation of 4-nitrophenol could be accomplished in the superior catalytic performance of RGO-Pt. The current strategy emphasizes a simple, fast and environmentally benign technique to generate low-cost gum waste supported nanoparticles with a commendable catalytic activity that can be exploited in environmental applications.

## 1. Introduction

Metal nanoparticles have gained considerable attention over the past few years owing to their remarkable physical and chemical properties—which is a stark contrast from their bulk counterparts. The properties arising due to the nano realm have made it possible for them to be used in a wide array of applications including catalysis [1,2], sensors [3], biomedicine [4,5,6], and drug delivery applications [7,8,9]. Over the years, different approaches have been developed for the synthesis of metal nanoparticles to obtain different sizes, structures, and morphologies. Conventional synthesis uses chemical reducing agents such as sodium borohydride (NaBH_4_), hydrazine, and dimethylformamide (DMF) to reduce metal cations to produce nanoparticles, with the inherent drawback that these chemicals are highly reactive and raise serious concerns regarding potential environmental impact and associated biological threats [10]. Extensive research has gone into developing new environmentally benign techniques to synthesize metal nanoparticles without the use of hazardous chemicals and solvents and preferably utilizing materials of biological origins, thus adhering to the principles of green chemistry. In addition to being eco-friendly, green synthesis offers several advantages over the conventional means, which include cost-effectiveness, simple experimental design, minimal to zero toxic chemicals, and byproducts [11] wherein precursors derived from various biological sources have been deployed successfully [12,13,14,15,16,17,18].

Biorenewable natural gums extruded from trees or extracted from seaweeds, and bacteria are a class of polysaccharides that have recently gained attention due to their ability to reduce metal salts to produce nanoparticles [19,20,21,22]. The complex polysaccharides and protein structures encompassing the gums provide a non-toxic alternative pathway to effectively reduce and stabilize the nanoparticles. Although numerous polysaccharides have been used in the greener assembly of nanoparticles, the use of gum kondagogu (GK) was much less explored; GK is a non-toxic, naturally occurring polysaccharide, readily available from the exudates of *Cochlospermum Gossypium* (Bixaceae family) tree [23]. GK contains a highly acidic sugar content, including galacturonic and glucuronic acids, which make up 52% of the total carbohydrates, while the remaining are neutral sugars such as glucose, galactose, rhamnose, and arabinose [24]. Upon toxicological evaluation, GK has been declared a non-toxic material, with potential applications as food additives [25] and can be an ideal reducing and stabilizing agent owing to its low cost, biorenewable nature, and availability of plentiful functional groups (hydroxyl, acetyl, carbonyl, carboxylic) with metal biosorption properties [16].

4-Nitrophenol (4-NP) is a toxic chemical often found in industrial and agricultural wastewaters. Various methods have been developed for the reduction and removal of 4-NP from these wastewaters and the reduction of 4-NP by NaBH_4_ is one of the simplest and most popular methods. However, the presence of a high kinetic barrier between 4-NP and BH_4_^−^ requires an effective catalyst in order for the reaction to proceed. Hence, researchers have been developing newer effective catalysts capable of assisting in the effective reduction of 4-NP. Additionally, the reduction of 4-NP by NaBH_4_ is often considered as a model reaction to assess the catalytic performance of novel catalysts.

Platinum (Pt) is one of the most extensively used catalyst elements in many chemical and electrochemical reactions [26], especially Pt nanoparticles with large surface area and greatly higher active sites, which have garnered significant attention due to their promising potential in the field of environmental and energy-related catalysis. With its unfilled 5d orbital and rich electronic structure, it is a well-established catalytic system in multifarious applications [27,28]. Thus, careful control and design of the surface and interface of the Pt nanoparticles with its in-depth analysis and comprehension of the structural aspects plays a pivotal role in optimizing the catalytic performance. Despite having an excellent catalytic ability, Pt nanoparticles are often associated with severe agglomeration, which reduces its catalytic performance [29]. To minimize agglomeration and to attain good dispersibility and stability, Pt nanoparticles are often deposited on support materials [30,31,32] that possess high specific surface area, abundant anchoring sites, high stability and high electric conductivity (for electrocatalytic applications). Carbon-based materials are the most commonly used ones for this purpose as they fulfill the requirement mentioned above [33,34,35,36,37,38,39]. Graphene, an atomically thin 2D material, suits all the above descriptions required for the deposition of Pt nanoparticles. Since its isolation in its pure form from graphite in 2004 [40], graphene has garnered significant attention for various applications. It can be synthesized by different methods including mechanical exfoliation [40,41], chemical vapor deposition [42,43], unzipping of carbon nanotubes [44,45], and synthesis on SiC [46,47]. Since these techniques are associated with high production costs, alternatively graphene oxide (GO) or reduced graphene oxide (RGO) can provide a low-cost scalable option and the presence of abundant oxygen functionalities offer anchoring sites for the metal nanoparticles to obtain NP decorated graphene sheets.

In the current study, we propose a simple, fast, inexpensive, and environmentally friendly process to deposit Pt nanoparticles on the graphene sheets. GO prepared by using the modified Hummers method [48] is conventionally reduced by hydrazine hydrate, NaBH_4_ or ethylene glycol [49,50,51,52]. In the present work, however, a green chemistry approach entails the simultaneous reduction of Pt salt and GO using GK waste to generate Pt nanoparticle adorning the RGO sheets. We have thoroughly characterized the obtained Pt nanoparticle decorated graphene sheets (Pt-RGO) using scanning electron microscopy (SEM), transmission electron microscopy (TEM), X-ray diffraction analysis (XRD), and Raman spectroscopy. Furthermore, the catalytic performance of the Pt-RGO catalysts was assessed in the hydrogenation of 4-nitrophenol (4-NP).

## 2. Results and Discussion

To optimize the process of NPs preparation on the GO surface, we carried out the synthesis at different reaction conditions and reagent concentrations (Details are presented in Table S1). From the results obtained, it was concluded that the optimum conditions were: 30 min reaction time, a temperature of 150 °C, and PtCl_4_ 1 mM. In other tested conditions, the reaction remains incomplete, which could be ascertained visually as the material does not completely precipitate (Figures S2 and S3).

The surface morphology of the thus prepared Pt-RGO was analyzed using SEM images. All the SEM images of the Pt-RGO from different metal salt concentrations (Figure S4) indicated folded structures on both, the surface and the edges, which suggests the typical morphology of few-layered RGO. It is evident from the images that at a lower PtCl_4_ concentration of 0.5 mM, only a few isolated nanoparticles can be seen. However, when the concentration was increased to 2 mM, a clear agglomeration of nanoparticles was discerned on the surface. At a concentration of 1 mM, a uniform finely dispersed Pt NPs deposition on the RGO surface can be detected.

**Figure 1:**
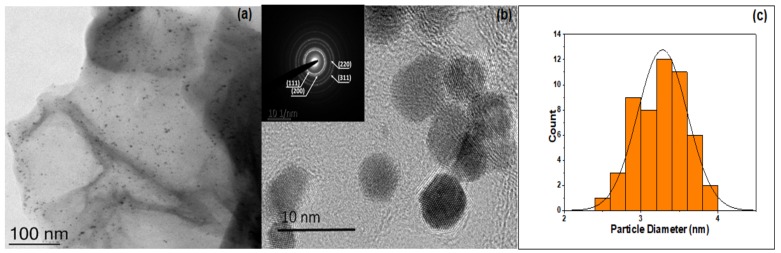
(**a**) TEM and (**b**) HRTEM image of the Pt-RGO; inset image corresponds to the SAED pattern of the Pt-RGO; (**c**) histogram depicting the size distribution of nanoparticles.

Furthermore, to analyze the morphology and to obtain insights into the particle size and distribution, TEM and HRTEM analysis were carried out (Figure 1) confirming that the GK had assisted in the formation and decoration of Pt nanoparticles on the RGO surface. The NPs were evenly dispersed throughout the surface with negligible agglomeration, which can be attributed to the presence of GK in the reaction medium.

The particles were mainly spherical shaped with a mean diameter of around 3.3 ± 0.6 nm. HRTEM images clearly show the lattice fringes and the *d* spacing was calculated to be around 0.22 nm corresponding to the (111) plane distance of face-centered cubic (fcc) crystalline Pt NP [53] with the SAED pattern showing diffraction rings corresponding to (111), (200), (220) and (311) reflections of fcc platinum crystals [53].

**Figure 2:**
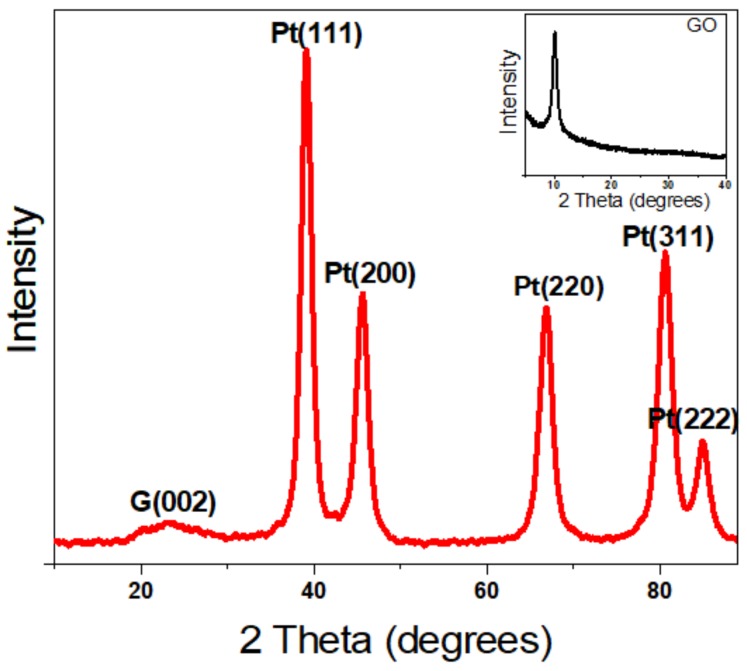
XRD pattern of the green synthesized Pt-RGO catalyst. The inset image is the XRD pattern of GO.

Furthermore, to analyze the crystal structure of the nanoparticles, the X-ray diffraction pattern of the Pt-RGO samples was recorded, as shown in Figure 2. GO before reduction shows a sharp diffraction peak at 2θ = 11°, which is a characteristic of GO and is associated with the chemical exfoliation of graphite [54], the appearance of a sharp plane (002) confirms the formation of exfoliated GO sheets [55]. Upon reduction, this peak is shifted to 23.5°, which affirms the formation of RGO [55] as evidenced by the XRD graphs of Pt-RGO (Figure 2). Additionally, five more major peaks were observed in Pt-RGO at 39.5, 45.6, 66.8, 80.7, and 85°, which corresponds to the (111), (200), (220), (311) and (222) planes of Pt, respectively [56]. These results are in accordance with the results obtained from SAED analysis and are consistent with the fcc structure of the platinum (JCPDS #040802), thus confirming the formation of crystalline Pt [56].

**Figure 3:**
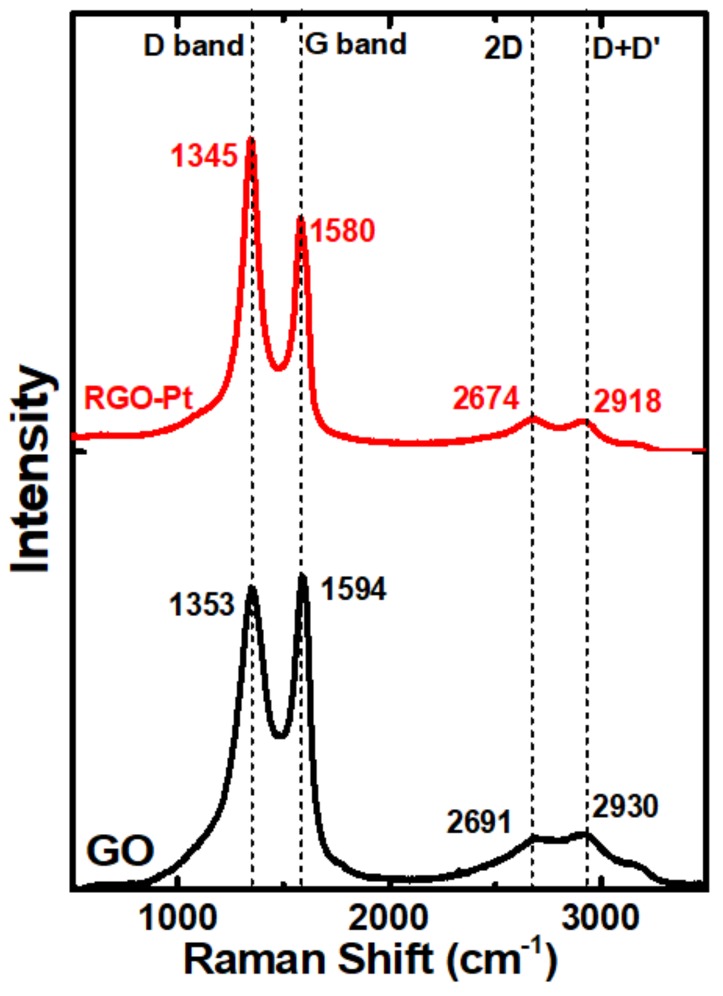
Raman spectra of GO and Pt-RGO.

Raman analysis of the Pt-RGO provides information on the nature of RGO and the formation of defects during the reduction of GO; Figure 3 displays the Raman spectra of GO and Pt-RGO catalysts. Before the reduction, GO show the major peaks at 1353 and 1594 cm^−1^ corresponding to D and G bands [57]. G band corresponds to the first-order scattering of the E_2_g mode and the D band arises due to the defects and asymmetrical breaking at the graphene edges. The lesser intensity bands at 2691 and 2930 cm^−1^ can be assigned to the 2D and D + D’ bands, respectively [58]. In the case of Pt-RGO, the same major and minor bands reappear, however, an obvious blue shift associated with RGO is observed. The D and G bands are shifted to 1345 and 1580 cm^−1^ respectively, while the 2D and D + D’ bands appear at 2674 and 2918 cm^−1^ respectively. The intensity ratio of D and G bands (I_D_/I_G_) for GO and Pt-RGO were found to be 0.96 and 1.34, respectively, and this increase in intensity indicated a reduction in the average domain size of the sp^2^ -hybridized carbon atoms upon reduction of exfoliated GO [59].

### Catalytic Reduction of 4-Nitrophenol

The catalytic performance of the prepared green catalyst was assessed by studying its ability to catalyze the reduction of 4-NP to 4-aminophenol. While 4-NP shows the maximum absorbance at ~317 nm, after the addition of sodium borohydride, this peak shifts to 401 nm due to the formation of 4-nitrophenolate, intermediate of 4-NP deprotonation. Due to the high energetic barrier, hydrogenation to 4-aminophenol does not proceed without a catalyst, which was confirmed by the unchanged intensity at 401 nm in time (data not shown). However, upon the addition of the Pt-RGO catalyst, the intensity at 401 nm continuously decreased and 4-aminophenol is formed. In an excess of NaBH_4_ (12 mM of NaBH_4_ and 0.12 mM of 4-NP) the catalytic activity of Pt-RGO was described by the pseudo-first-order kinetic model [60]; the experiment being performed for four different Pt-RGO concentrations (0.0012 to 0.01 g/L).

The absorbance *A* can be considered linearly proportional to the concentration of *C* of 4-NP, and A/A_0_ is proportional to *C*/*C*_0_, and thus the rate constant of the pseudo-first-order kinetic reaction can be calculated using Equation (1).

(1) $$ln\left(\frac{A_{t}}{A_{0}}\right)=-kt.$$

**Figure 4:**
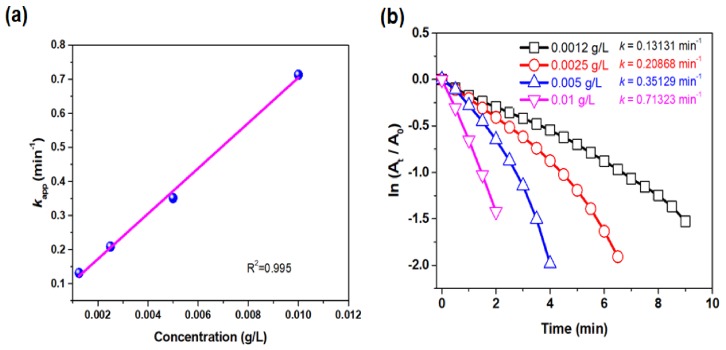
(**a**) *k*_app_ (min^−1^) vs. concentration (g/L) of Pt-RGO used, (**b**) plot of ln (A_t_/A_0_) versus reaction time for different Pt-RGO concentrations (0.0012 to 0.01 g/L) for the reduction of 4-NP.

By following Equation (1), the rate constants (*k*_app_) were determined to be 0.13, 0.20, 0.35 and 0.71 min^−1^ for the Pt-RGO catalyst concentration of 0.0012, 0.0025, 0.005 and 0.01 g/L, respectively (Figure 4b). The performance of the catalyst is linearly proportional to the concentration of catalyst used, hence, upon increasing the concentration of the catalyst, the *k*_app_ increased as well. This can be explained by the fact that when the concentration of the catalyst increased, the total surface area increased along with the number of active sites available for the catalytic reactions (Figure 4a) corroborating the earlier reports on the linear increase of *k*_app_ value with the increase in catalyst concentration. Lara et al. [61] reported an increase of *k*_app_ when the palladium-based catalyst concentration was increased. Similarly, Kästner and Thünemann [62] reported in their work that increasing the catalyst concentration (Ag NP) could increase the *k*_app_ in a linear way.

4-NP is one of the most used compounds for testing the catalytic activity of the catalysts; therefore, it should be easy to compare the catalytic performance of different catalysts. Further, to emphasize on the good catalytic performance of the Pt-RGO green catalysts, we have tabulated the *k*_app_ values of different studies in comparison with our study in Table 1.

## 3. Materials and Methods

### 3.1. Chemicals and Materials

GK, a grade III non-edible gum waste (Figure S1), was collected from Girijan Co-operative Corporation, Hyderabad, India. Graphite with particle size < 50 μm was purchased from Merck. PtCl_4_ (96%), sodium borohydride (98%), 4–nitrophenol (ReagentPlus > 99%) were procured from Sigma Aldrich. Sulfuric acid (96 wt%), hydrochloric acid (35 wt%), hydrogen peroxide (30 wt%), potassium permanganate, sodium nitrate was procured from Penta, Czech Republic. Deionized water (18.2 MΩ·cm^−^^1^, ELGA, Veolia Water, Marlow, UK) was to carry out the experiments.

### 3.2. Synthesis of Pt-RGO Nanoparticles

GK was thoroughly cleaned by three washings with distilled water. To remove water and residual moisture from the gum samples, they were dried overnight in an oven at 80 °C. Completely dried gum samples were then powdered using a high-speed mechanical blender and then sieved using a mesh size of 250 µm to obtain a uniform fine powder.

Graphene Oxide (GO) was produced from graphite flakes using the modified Hummers method [48,67] In a typical process, 69 mL of concentrated sulfuric acid along with 1.5 g of sodium nitrate was taken in a two-necked round-bottomed flask to which 3 g of graphite powder was added gradually over 30 min period under constant stirring. The flask was then kept on an ice bath and 9 g of potassium permanganate powder was added very slowly for over 30 min; the temperature of the reaction was maintained so that it does not exceed 20 °C. After the addition, the ice bath was removed and the mixture was stirred at 35 °C for 12 h. Then, 138 mL of deionized water was poured into the solution, which raised the temperature up to 98 °C and turned the solution dark brown in color. The stirring was continued for another 2 h, after which 30 mL of 30% hydrogen peroxide was added to convert MnO_2_ to soluble MnSO_4_; the color of the mixture changed to golden yellow and the reaction was continued for another 30 min. Then, the mixture was washed with 5% HCl several times to remove sulfate salts. The product was made alkaline by washing it with 5% solution of sodium carbonate and the ensuing solid product was isolated by filtration and dried in an oven for 24 h at 75 °C.

Pt-RGO was prepared using the one-pot synthesis strategy via simultaneous reduction of both PtCl_4_ salt and GO by GK waste. Briefly, GO dispersed in water, was added into PtCl_4_ water solution (0.25–2 mM) under constant stirring such that the final GO concentration is 1 g/L. Then, the mixture was sonicated for 30 min to enable fine dispersion. A 2.5 mL of the above mixture was mixed with 2.5 mL GK solution (processed GK via deacetylation protocol; stock solution = 20 g L^−1^) [24] under vigorous stirring in a sealed ampoule. The reaction was allowed to proceed for 30 min under heating (120–150 °C) in a dry bath, when reduced graphene oxide precipitated as a black solid; solution color became clear, as opposed to the initial light yellow color of PtCl_4_, indicating the complete reduction of the metal salt. The ensuing product was isolated by centrifuging and washed with deionized water. Finally, the product was dried in an oven overnight to remove any residual moisture. The synthesis was conducted under different reaction conditions by varying time, temperature and concentration of PtCl_4_ to optimize the synthetic protocol.

### 3.3. Characterization Techniques

Scanning Electron Microscopy (SEM) of the samples was assessed on UHR FE-SEM Carl Zeiss ULTRA Plus, Germany, operating at 0.5–2.5 kV acceleration voltage. Further, to analyze the morphology and selected area electron diffraction (SAED) patterns field emission transmission electron microscopy (FE-TEM) JEM-2100F, JEOL Ltd., Japan, operating at 200 kV, was used. The particle size distribution was calculated by measuring 50 particles using HRTEM images by using ImageJ. The obtained micrographs were processed by using the digital image processing software Gatan Digital Micrograph. The crystallographic structures of the material were determined using X-ray diffraction (XRD) spectroscopy, which was performed on Bruker, AXS/8, Berlin, Germany and the diffraction spectrum over 2θ range of 0 to 80° was obtained using Cu-Kα radiation (40 kV, 60 mA) to identify the crystal phase and composition. Raman Spectroscopy was carried out using a Raman DXR microscope (Thermo Fisher, Waltham, MA, USA), with a laser excitation wavelength of 514 nm on an Argon laser with 1 cm^−1^ spectral resolution. The spectra were recorded at ambient conditions in the range 1000–3000 cm^−1^. UV-vis spectroscopy was performed on Hach Lange DR 3900 UV-vis spectrophotometer, UK using 1 cm quartz cuvettes.

### 3.4. Catalytic Reduction of 4-Nitrophenol (4-NP)

The catalytic prowess of Pt-RGO catalysts was evaluated in hydrogenation of 4-NP to 4-aminophenol using NaBH_4_, where the procedure was followed on the lines previously reported by Baruah et al. [68]. Initially, 24 µL of 4-NP (5 mM) was taken in an Eppendorf tube to which the prerequisite amount of Pt-RGO was added (0.0012 to 0.01 g/L). To this mixture, 120 µL of NaBH_4_ (0.1 M) was added followed by the addition of DI water to adjust the volume to 1 mL. This solution mixture was thoroughly mixed and immediately transferred to a clean quartz cuvette (1 cm path length), and the absorbance was recorded continuously over regular time intervals using a UV-Vis spectrometer.

## 4. Conclusions

A one-pot co-reduction method was uncovered successfully to obtain RGO sheets decorated uniformly with Pt nanoparticles using biorenewable GK waste as a reducing agent. Even though GK has been used in the synthesis of metal nanoparticles, its ability to simultaneously reduce GO and metal salts has not been studied until now, to directly generate nanocomposite from a readily available and eco-friendly precursor. Furthermore, the proposed synthesis is fairly expeditious as compared to other reported methods [63,69,70] GK has not only successfully reduced Pt salt and GO simultaneously, but also assisted in the fine dispersion of Pt on the RGO surface; hydroxyl groups on the GO surface provide anchoring sites for the deposition of Pt nanoparticles. The obtained Pt-RGO nanoparticles were further analyzed by SEM, TEM, XRD, and Raman spectroscopy, where morphological studies showed that the ensuing nanoparticles were spherical in shape with an average particle size of 3.3 ± 0.6 nm and the Raman studies and XRD results established the successful reduction of GO by GK. In addition, XRD results indicated the formation of crystalline Pt nanoparticles with fcc geometry. The Pt-RGO thus obtained, displayed good catalytic performance in the hydrogenation of 4-nitrophenol, wherein the hydrogenation of 4-nitrophenol occurred in 2 min reaction time with a rate constant (*k*_app_) of 0.71 min^−1^. The superior catalytic performance of the Pt-RGO catalyst can be attributed to the synergistic effect of Pt nanoparticles anchored on the RGO surface. The simple and easy synthesis of Pt-RGO follows greener synthesis protocols and offers attractive possibilities to be used in the production of efficient green catalysts for the reduction of toxic and hazardous chemicals in various fields.

## Figures and Tables

**Table 1:** Comparison between different catalysts with our present study.

Catalysts	4-NP Concentration (mM)	NaBH_4_ Concentration (mM)	Reducing/Supporting Material	Reaction Time (min)	Rate Constant (*k*_app_) min^−1^	Reference
Pt-RGO	0.12	12	GK	2	0.71	This work
Pd-RGO/GA	5	0.5	Gum Arabic	5	0.12	[63]
Au_53_Pd_47_/Graphene nanosheets	0.05	5	-	3.5	0.86	[64]
GPt-RGONPs	0.024	0.024	Guar gum	240	0.42	[21]
AgNPs@MWCNTs	0.1	5	Chitosan	5	0.47	[65]
XG/Ag	1 × 10^−4^	0.1	Xanthan gum	1440	0.90	[20]
LrGO-Ag_20_Au_80_	9.6 × 10^−5^	0.1	*Cetraria Islandica*	1.7	0.45	[66]

## Supplementary Materials

The following are available online at https://www.mdpi.com/1420-3049/24/20/3643/s1, Figure S1: Image of the gum kondagogu (grade III-non-edible gum). Figure S2: SEM images of Pt-RGO with different PtCl4 concentration. (a) 0.25, (b) 0.5, (c) 1, and (d) 2 mM. Figure S3: Images of the final products of different temperature (I) 120, (II) 130, (III) 140, and (IV) 150 °C. Figure S4: Images of the final products of different time (I) 15, (II) 30, and (III) 60 min. Figure S5: EDX profile of Pt-RGO. Figure S6: Plot of ln (At/A0) versus reaction time of G-Pt of varying concentrations (a) 0.0012, (b) 0.0025, (c) 0.005 and (d) 0.01 g/L. Table S1: Different reaction conditions and concentrations for the optimization of the reaction conditions.
