# Supplementary material for: Gum Kondagoagu/Reduced Graphene Oxide Framed Platinum Nanoparticles and Their Catalytic Role
Source: Molecules. 2019 Oct 9;24(20):3643. doi: 10.3390/molecules24203643 (PMC6832613; doi:10.3390/molecules24203643)
Supplement: Supplementary file 1 [file molecules-24-03643-s001.pdf]

molecules

*Supplementary Information*

# Gum Kondagogu/Reduced Graphene Oxide Framed Platinum Nanoparticles and Their Catalytic Role

**Abhilash Venkateshaiah [1], Daniele Silvestri [1], Rohith K. Ramakrishnan [1], Stanislaw Wacławek [1], Vinod V.T. Padil [1,*], Miroslav Černík [1,*] and Rajender S Varma [2,*]**

[1] Department of Nanomaterials in Natural Sciences, Institute for Nanomaterials, Advanced Technologies and Innovation (CXI), Technical University of Liberec (TUL), Studentská 1402/2, 46117 Liberec 1, Czech Republic

[2] Regional Centre of Advanced Technologies and Materials, Department of Physical Chemistry, Faculty of Science, Palacký University in Olomouc, Šlechtitelů 27, 78371 Olomouc, Czech Republic

* Correspondence: vinod.padil@tul.cz (V.V.T.P.); miroslav.cernik@tul.cz (M.Č.); Varma.Rajender@epa.gov (R.S.V.)

**Supporting Information Content:**

Six pages (including the cover page).

**Table S1.** Different reaction conditions and concentrations for the optimization of the reaction conditions.

**Figure S1.** Image of the gum kondagogu (grade III-non-edible gum)

**Figure S2.** Images of the final products of different temperature (I) 120, (II) 130, (III) 140 and (IV) 150 °C

**Figure S3.** Images of the final products of different time (I) 15, (II) 30 and (III) 60 min.

**Figure S4.** SEM images of Pt-RGO with different $PtCl_4$ concentration. (a) 0.25, (b) 0.5, (c) 1 and (d) 2 mM.

**Figure S5.** EDX profile of Pt-RGO.

**Figure S6.** Plot of ln ($A_t/A_0$) versus reaction time of G-Pt of varying concentrations (a) 0.0012, (b) 0.0025, (c) 0.005 and (d) 0.01 g/L

**Table S1.** Different reaction conditions and concentrations for the optimization of the reaction conditions.

| GO concentration (g/L) | $PtCl_4$ concentration (mM) | Temperature (°C) | Time (min) | Result |
|---|---|---|---|---|
| 1 | 1 | 120 | 30 | Incomplete |
| 1 | 1 | 130 | 30 | Incomplete |
| 1 | 1 | 140 | 30 | Incomplete |
| 1 | 1 | 150 | 30 | Complete |
| 1 | 1 | 150 | 15 | Incomplete |
| 1 | 1 | 150 | 30 | Complete |
| 1 | 1 | 150 | 60 | Complete (no further change) |
| 1 | 0.25 | 150 | 30 | Complete |
| 1 | 0.5 | 150 | 30 | Complete |
| 1 | 2 | 150 | 30 | Complete |

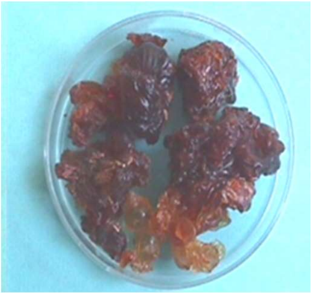

**Figure S1:** Specimen of the non-edible grade III gum kondagogu (GK)

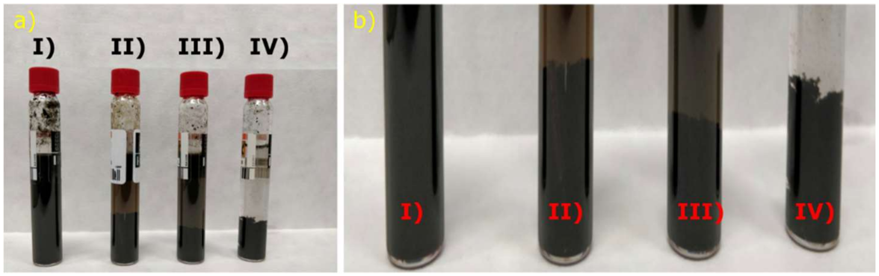


**Figure S2.** Images of the final products of different temperature (**I**) 120, (**II**) 130, (**III**) 140 and (**IV**) 150 °C

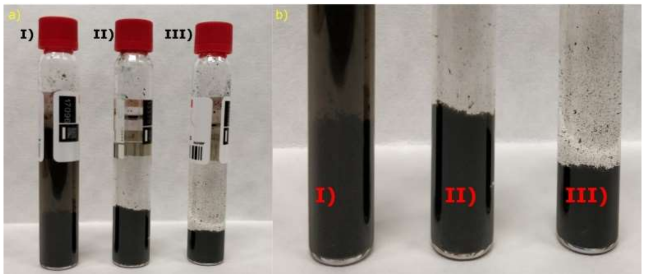


**Figure S3.** Images of the final products of different time (**I**) 15, (**II**) 30 and (**III**) 60 min.

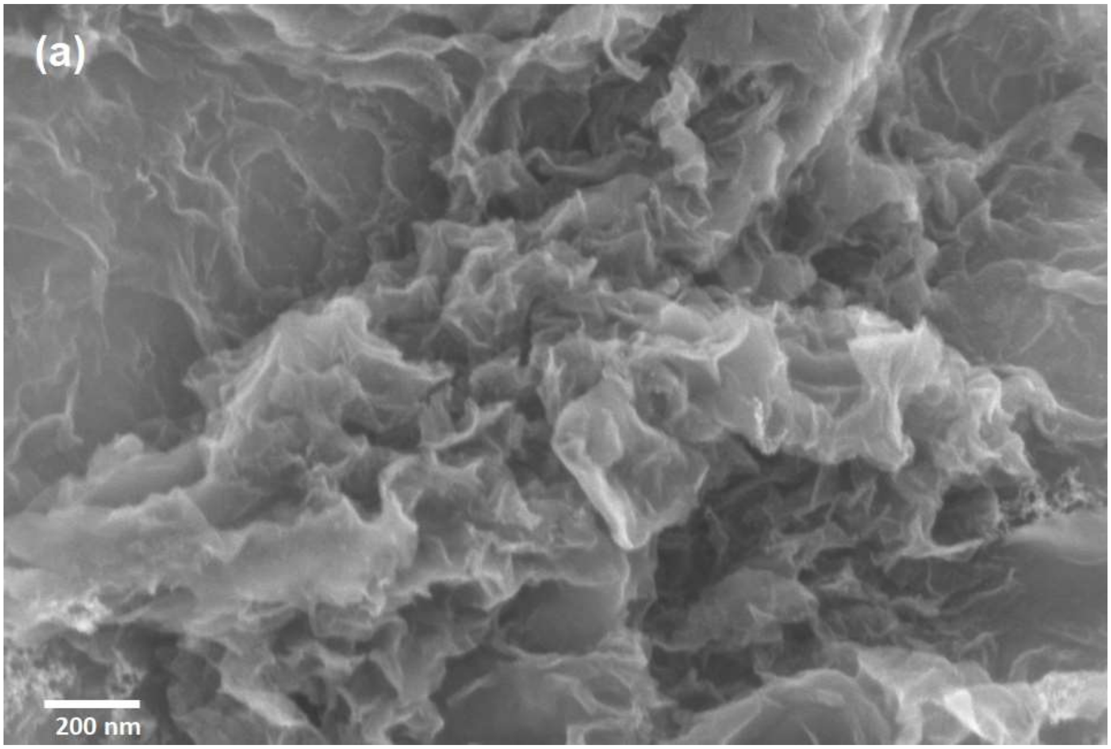

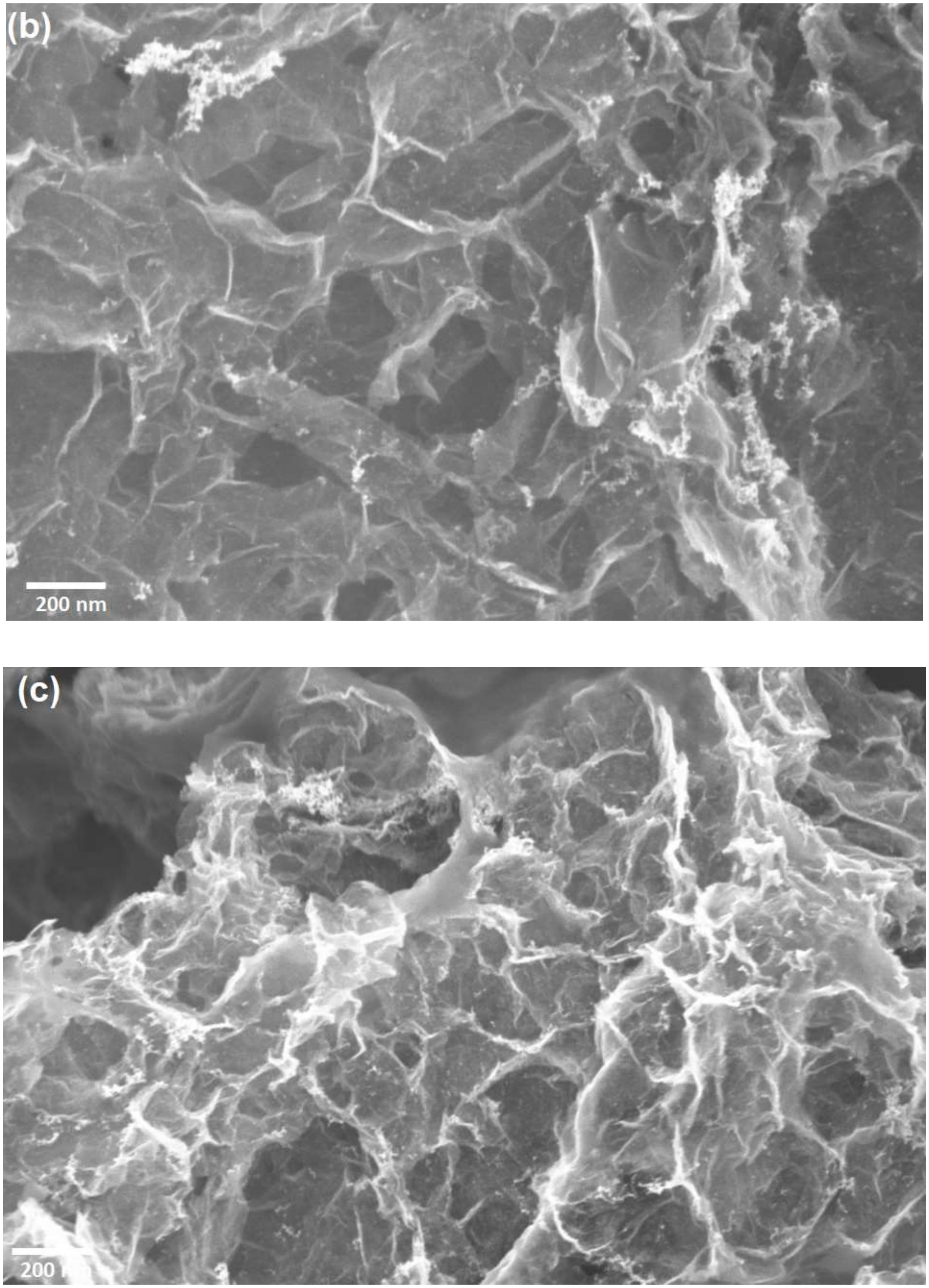
(b)
200 nm
(c)
200

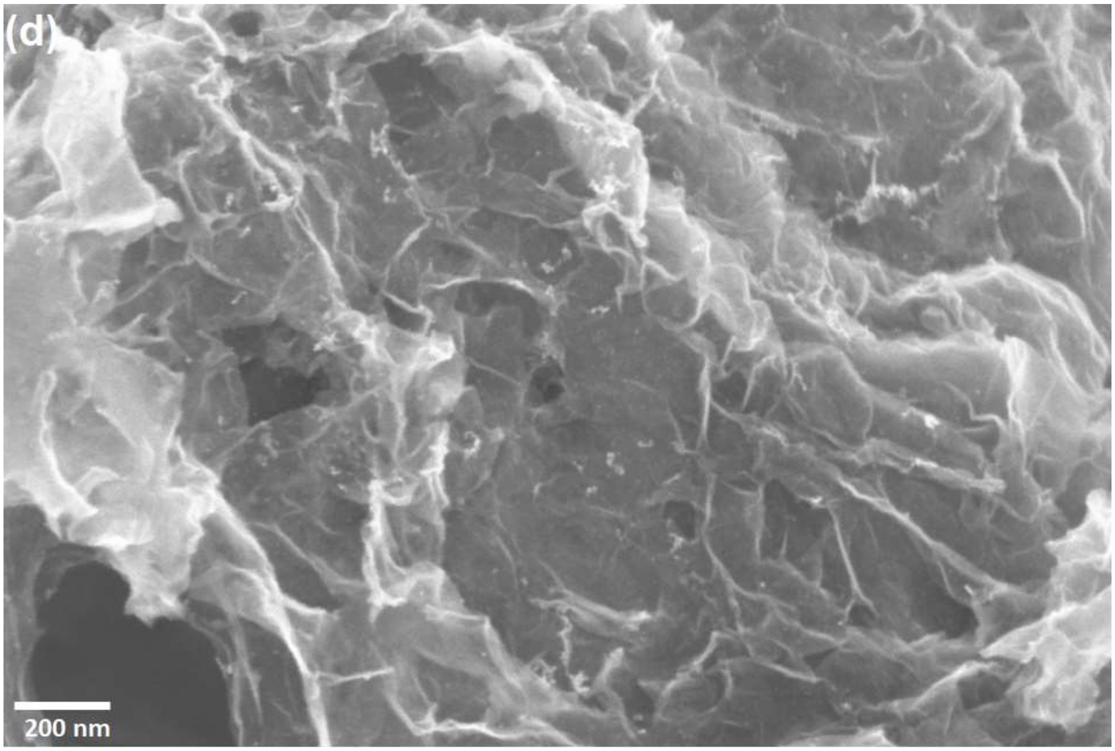


**Figure S4.** SEM images of Pt-RGO with different $PtCl_4$ concentration. (**a**) 0.25, (**b**) 0.5, (**c**) 1 and (**d**) 2 mM

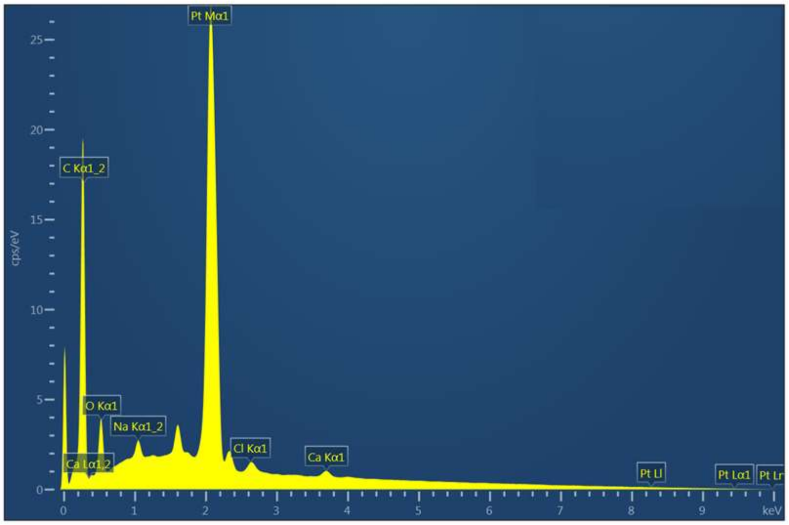


**Figure S5.** EDX profile of Pt-RGO.

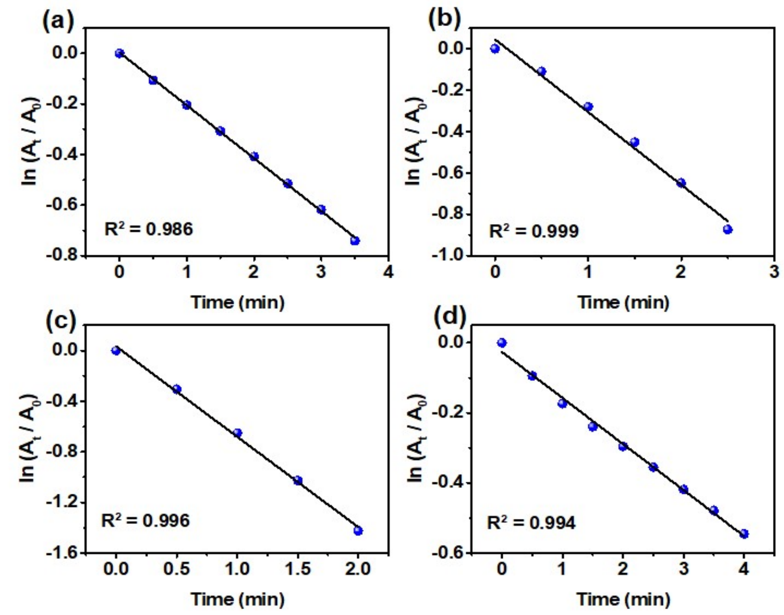


**Figure S6.** Plot of ln ($A_t/A_0$) versus reaction time of G-Pt of varying concentrations (**a**) 0.0012, (**b**) 0.0025, (**c**) 0.005 and (**d**) 0.01 g/L.
